# Exploring the preferred integration approach for HIV, diabetes and hypertension care and associated barriers and facilitators in Central Tanzania: An exploratory qualitative study

**DOI:** 10.1371/journal.pgph.0003510

**Published:** 2024-07-24

**Authors:** Tiffany E. Gooden, Mkhoi L. Mkhoi, Lusajo J. Mwalukunga, Mwajuma Mdoe, Elizabeth Senkoro, Stephen M. Kibusi, G. Neil Thomas, Krishnarajah Nirantharakumar, Semira Manaseki-Holland, Sheila Greenfield

**Affiliations:** 1 Institute of Applied Health Research, University of Birmingham, Birmingham, United Kingdom; 2 Department of Microbiology and Parasitology, University of Dodoma, Dodoma, Tanzania; 3 Department of Public Health, University of Dodoma, Dodoma, Tanzania; 4 Ifakara Health Institute, Ifakara, Tanzania; Tribhuvan University Institute of Medicine, NEPAL

## Abstract

Timely diagnosis and management of diabetes and hypertension among people living with HIV (PLWH) is imperative; however, many barriers exist within the current model of care for these comorbidities. We aimed to understand how HIV, diabetes, and hypertension care should be delivered and the associated barriers and facilitators for the preferred delivery approach. We conducted semi-structured interviews with 16 PLWH with comorbidities of diabetes and/or hypertension (referred to hereafter as non-communicable diseases [NCDs]), 10 healthcare professionals (HCPs) that provide care for NCDs, and 10 HCPs that provide care for HIV. Participants were recruited from two healthcare facilities in Dodoma, Tanzania and interviewed in Swahili. Interviews were audio recorded, transcribed verbatim and translated into English. We used the differentiated service delivery building blocks as a framework to determine where, who, what and when care should be provided. We applied the Theoretical Domains Framework (TDF) to HCP transcripts to determine barriers and facilitators for the preferred integration approach. There was a consensus among participants that all care for NCDs should be provided for PLWH at HIV clinics (known as care and treatment centres [CTCs]) by either CTC doctors or NCD specialists. Participants preferred flexible follow-up care for NCDs and for it to be aligned with HIV follow-up appointments. The main barriers were mapped to the TDF domains of environmental context and resources, and social influences; the former included the lack of NCD medications, NCD diagnostic equipment, space, staff and guidelines whereas the latter included negative influences from peers and traditional healers. Several facilitators were mentioned regarding CTC HCPs’ knowledge, skills, optimism and beliefs regarding their capabilities to care for PLWH with NCDs. The preferred integration approach should be tested, utilising the enabling factors described. The barriers described must be addressed with or without integration to achieve optimal care for PLWH with NCDs.

## Background

In 2022 there were 39 million people living with HIV (PLWH), of which 1.3 million people were newly diagnosed [1]. HIV burden is highest in sub-Saharan Africa where 1 in 30 adults are living with HIV and 66% of all PLWH reside [1]. With extended access to and uptake of effective antiretroviral therapy (ART), the life expectancy is increasing for PLWH, [2] putting them at increased risk for developing age-related comorbidities. Additionally, the toxicity and duration of ART, and persistent inflammation and viral presence (even when virally supressed) has multiple and complex biological impacts such as microbial translocation and increased pro-inflammatory cytokines [3]. These factors result in an increased risk for hypertension and diabetes, and other non-communicable diseases (NCDs) among PLWH compared to people without HIV of the same age [4]. In many parts of the world, HIV and NCD care are provided in separate clinics, often with limited communication or information exchange between the healthcare professionals (HCPs) managing HIV and HCPs managing NCDs.

**Fig 1:**
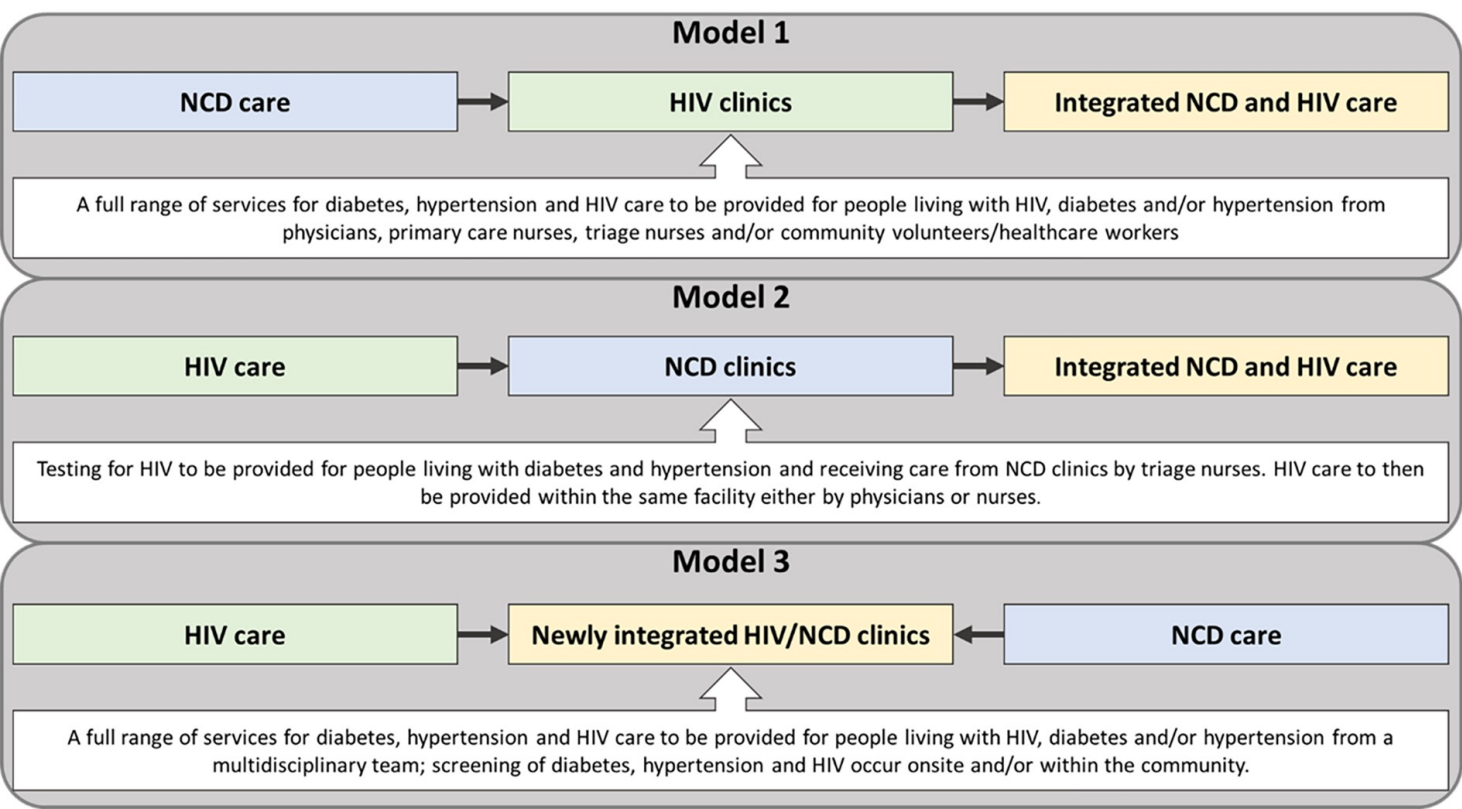
The three approaches of integrated care for diabetes, hypertension and HIV care, recreated from findings by Duffy et al. [8].

Separate clinics for HIV and chronic NCDs like diabetes and hypertension can disrupt continuity of care, and increase the risk of polypharmacy and drug-drug interactions; thus, patient safety and satisfaction has been shown to be negatively impacted by fragmented care for co-existing chronic conditions [5]. This has also been found in Tanzania where care for HIV, diabetes and hypertension are fragmented with limited efforts to achieve continuity of information between and within healthcare facilities [6]. Many barriers have been reported for this model of care delivery in Tanzania and many related to organisational and healthcare system factors that integration of care may overcome [6]. Indeed, the World Health Organisation (WHO) added a new recommendation for the integration of diabetes, hypertension and HIV care in the 2021 guidelines on the delivery of HIV care with the caveat that more research is needed to understand the best way to achieve effective integration [7]. Integration of services require changes to practice and structure of healthcare delivery which involve individual and collective behaviour changes and adaptations. Although models have been proposed (Fig 1), [8] it is important to understand and address the perspectives of people directly affected by any proposed changes (i.e. HCPs and PLWH) and understand potential barriers or facilitators for implementation.

To our knowledge, ten qualitative studies have been conducted in Africa aimed at seeking views on integrated HIV, diabetes and/or hypertension care: four in Uganda, [9–12] three in South Africa, [13–15] two in Nigeria, [16, 17] and one in Tanzania [18]. However, most studies (n = 7) assessed the barriers and facilitators after integration of care was introduced instead of to inform how integration should be achieved; one study reported the barriers and facilitators of a proposed integrated care programme. Of the remaining four, three were conducted outside South Africa which has different infrastructure and resources available than other sub-Saharan African settings and; one from Nigeria [16] only included stakeholders from national or regional organisations and did not include PLWH nor HCPs that would be involved in receiving or delivering integrated care. The remaining studies from Uganda [10] and Tanzania [18] were part of one large trial where a ‘one-stop shop’ for people to receive care for diabetes, hypertension or HIV was tested; the interviews conducted with HCPs and PLWH were conducted before and after integration. Prior to integration, both studies found that HCPs thought positively about integration; however, people without HIV had concerns about attending a clinic with PLWH and PLWH had concerns about their confidentiality in an ‘one-stop shop’ from the Tanzanian study. Neither study appeared to ask participants about their preferred integration approach to determine context-specific preferences and barriers and facilitators for adapting and effectively implementing the preferred integrated care system.

**Fig 2:**
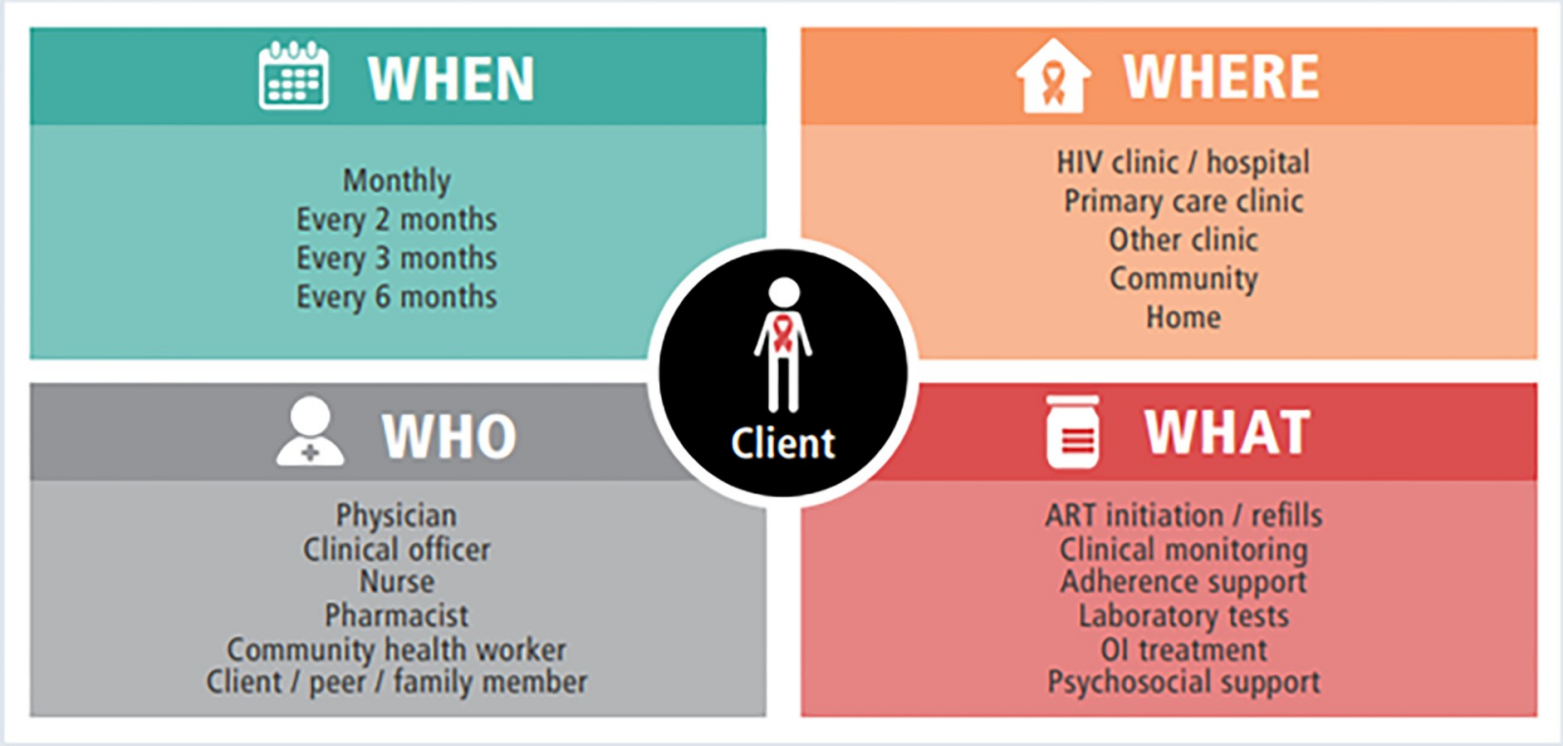
The differentiated service delivery (DSD) model developed by the World Health Organisation. Source: World Health Organization. Updated recommendations on service delivery for the treatment and care of people living with HIV: World Health Organization; 2021 [7].

As per the differentiated service delivery (DSD) model recommended by the WHO (Fig 2) [7] and implemented in many African settings, including Tanzania, it is important to understand how to integrate care for HIV, diabetes and hypertension based on the preferences of 1) where care should be provided, 2) who should provide care, 3) what care should be provided, and 4) when care should be provided. This model seeks to deliver care that is person-centred and adapted to meet local needs and resources. [7] We aimed to explore preferences on the integration of care for HIV, diabetes and hypertension in Tanzania using the building blocks of the DSD model (where, who, what and when) as a theoretical framework. This framework was chosen given its roots in a patient-centred approach to ensure care delivery meets the health needs and preferences of PLWH, but also the professional needs and preferences of HCPs [7]. We also aimed to understand whether any barriers or facilitators may exist for implementation of the preferred integration approach using the Theoretical Domains Framework (TDF) [19, 20]. The TDF was chosen based on its adaptability, multidimensional elements and comprehensive and systematic approach for identifying and categorising factors that influence HCP behaviour and clinical practice [19].

## Methods

### Study design

We conducted a qualitative study using semi-structured interviews (SSIs) [21] with HCPs and PLWH in Dodoma, Tanzania from 20^th^ October 2022 to 11^th^ November 2022.

### Setting

Dodoma is the capital and governmental hub of Tanzania; however, most of the Dodoma region is rural with a population of approximately two million people [22]. Participants were recruited from Dodoma Regional Referral Hospital and Makole Health Centre located within Dodoma city centre due to the high number of PLWH registered at these facilities; they see the largest number of PLWH in the Dodoma region (over 9000 PLWH combined at the time of data collection) and has the largest catchment area in Dodoma. Both facilities have a care and treatment centre (CTC) that provides free HIV care to PLWH and an outpatient department (OPD) that provides care to people with NCDs, including diabetes and hypertension. HIV care, including diagnostic tests, ART and CD4 and viral load tests are free to all Tanzanian citizens whereas NCD care is only free to the elderly and those with insurance. In 2018, only 33% of the population was covered by one of the two public health insurance schemes [23].

### Study participants

Nurses and doctors working in the CTC or OPD were eligible for inclusion. HCPs were excluded if they had less than six months of work experience. PLWH with a comorbidity of diabetes and/or hypertension, diagnosed after HIV and between three months and five years prior to the interview were eligible. The CTC officer in charge from both healthcare facilities recruited the participants. We aimed to recruit 10 CTC HCPs, 10 OPD HCPs and 16 PLWH informed by existing literature; [24] with this, we aimed to reach a consensus among participant groups on preferences of care and saturation among HCPs related to barriers and facilitators [25, 26]. We aimed to split each participant group evenly across the two facilities and recruit at least eight PLWH with diabetes and eight with hypertension. CTC officers in charge were instructed to recruit purposively based on sex and age due to the relevancy of these characteristics to the research question (i.e. preferences of care often differ by sex and age) and feasibility (i.e. this information was readily available to the recruiting CTC officers) [27]. Participants were recruited by phone at the Dodoma Regional Referral Hospital and in person at Makole Health Centre. HCPs were recruited in person, on non-clinic days.

### Data collection

Topic guides for HCPs and PLWH were developed in English then translated into Swahili. Topic guides for PLWH and HCPs included questions regarding their preferences for delivery of HIV, diabetes and hypertension care. The HCP topic guide had additional questions that were developed using the TDF to identify the barriers and facilitators for their preferred delivery of care [19, 20]. The TDF was designed to identify determinants of behaviour for implementation of an intervention [19]. There are 14 domains in the TDF; however, we identified the following eight as being relevant for our study, given the local context and the behaviour we wanted to investigate was non-specific (i.e. a specific integration intervention was not questioned, instead we queried the delivery of care that each participant recommended): [19, 20] 1) environmental context and resources, 2) knowledge, 3) skills, 4) professional role, 5) optimism, 6) memory, attention and decision processes, 7) beliefs about capabilities and 8) social influences. The research team at the University of Birmingham worked with collaborators at the University of Dodoma to co-develop the topic guides after assessing the questions for culturally and contextually appropriate wording, terminology and theory.

Research assistants (MM and LJM), both PhD students at the University of Dodoma conducted the interviews in Swahili in a quiet private room within the CTCs. MM (female) and LJM (male) both have experience with collecting qualitative data and were trained for the current study. MM has a degree in midwifery and LJM has a degree in nursing; neither had a prior relationship with any of the participants. All interviews were audio recorded. TEG (female), a research fellow and PhD candidate in global health from the University of Birmingham was present for half of the interviews as an observer, split across each participant group and between the two research assistants to reduce interview effect. This work forms a chapter of TEG’s PhD thesis. All participants were financially compensated for their time. On average interviews took around 40 minutes. Interviews were transcribed directly into English due to limited resources and time; TEG created the English transcripts as MM and LJM (Tanzanian nationals fluent in Swahili and English) verbally translated each audio recording verbatim and concurrently clarified any contextual or cultural uncertainties for later interpretation.

### Analysis

Data was initially analysed inductively using the Framework Method [28] with open coding applied to all transcripts line-by-line. Codes were recorded in separate matrices for HCPs and PLWH using Excel. This method was used to enable easy identification of relevant codes regarding preferences of care and any similarities or differences in preferences within and between participant groups (PLWH and HCPs). The codes were used to identify the preferences of HCPs and PLWH on how care for HIV, diabetes and hypertension should be integrated (primary analysis for the current study). We used the building blocks of the DSD model i.e. who, what, where and when care should be provided as the framework for the analysis [7]. TEG manually conducted the initial coding and applied the DSD building blocks to the codes. An expert in qualitative methods and Professor of Medical Sociology (SG) independently coded the first four HCP transcripts to ensure reliability. All findings were discussed and agreed with the research team, including MM and JLM who conducted the interviews.

After preferences of care were identified, TEG re-read the HCP transcripts line-by-line, and using a deductive content analysis [29] manually mapped barriers and facilitators for integrating diabetes, hypertension and HIV care to the relevant eight TDF domains [19]. A content analysis was deemed most appropriate with the TDF domains applied as themes to specifically identify the related concepts regarding barriers and facilitators. Only barriers and facilitators for integrating care based on the preferences identified from the primary analysis were mapped.

### Ethics approval and consent to participate

Ethical approval was received by the Institutional Research Review Ethics Committee at the University of Dodoma (reference number MA.84/261/02/07). No deviation to the study protocol was made after ethical approval. Approval from each hospital’s medical officer in charge was sought and received before any recruitment commenced. Before each interview commenced, participants were briefed on the aim of the study, the benefits and risks of their participation, the voluntary nature of the interview and terms for anonymity and withdrawal. All participants provided written consent to take part and provided further verbal consent prior to starting the audio recorder. Participants were given sufficient time to review the participant information sheet and ask any questions before providing consent. Interviewers read out loud the participant information sheet and consent form to illiterate participants prior to obtaining written consent by thumbprint.

## Results

A total of 36 interviews were conducted (10 CTC HCPs, 10 OPD HCPs and 16 PLWH). Half of the CTC and OPD HCPs were from Dodoma Regional Referral Hospital and the remainder from Makole Health Centre; 10 PLWH were recruited from the former and six from the latter. Table 1 summarises the participant demographics.

**Table 1:** Participant characteristics.

Characteristics	CTC HCPs N = 10	OPD HCPs N = 10	PLWH N = 16
Sex			
Males	4	4	6
Females	6	6	10
Age			
20–30	4	1	0
31–40	1	6	0
41–50	2	1	3
51–60	2	2	8
61+	1	0	5
Highest level of education			
No formal education	0	0	3
Primary education	0	0	11
Secondary education	0	0	1
University degree	10	10	1
Profession			
Doctor	6	8	--
Nurse	4	2	--
Duration of experience			
< 1 year	3	4	--
1–10 years	5	4	--
> 10 years	2	2	--
Comorbidity			
Diabetes	--	--	4
Hypertension	--	--	8
Diabetes and hypertension	--	--	4

A dash indicates data that was not relevant to collect.

### Preferences for integrated care

There was consensus from participants that care for HIV, diabetes and hypertension should be integrated. An exception to this was one CTC doctor who felt separate clinics was best for delivering high quality care because it enabled PLWH to receive specialised care for each condition. However, the participant acknowledged the disturbance separated care likely causes PLWH and after further probing, it appeared the participant was reluctant towards the idea of integration due to the additional workload it may bring to the CTC. We describe below where, who, what and when care for HIV, diabetes and hypertension should be provided within an integrated system, from the perspective of HCPs and PLWH.

### Where should care be provided?

Most HCPs and PLWH stated that care for diabetes and hypertension should be provided alongside HIV care at CTCs. It was stated that PLWH would be fearful of others finding out about their HIV status at the OPD or within the community if all care was provided by OPD HCPs or community healthcare workers, respectively. Many PLWH said that they are close with CTC HCPs because they have been receiving care from them for many years; they said they trust CTC HCPs because they do not stigmatise them and they give them hope and good advice. One PLWH mentioned that they preferred going to the CTC because they like speaking with fellow PLWH and sharing their experiences with one another. HCPs said that the CTC has more clinic days and more flexible hours compared to the OPD, providing more opportunity for PLWH to receive timely and essential care for comorbid diabetes and hypertension.

“these services should be a one stop shop, that when the patient comes to the CTC it would be good for them to receive all the care here. Because if you start telling the patient to go here go there, it’s disturbing to the patients because going out of CTC to look for care to another department, the patient may feel that people know he has HIV virus. So the patient normally looks worried.” (CTC nurse P8)

“at the CTC they have clinic everyday so there should be a permanent person that deals with NCDs to HIV clients and other comorbidity diseases to HIV clients.” (OPD doctor P11)

“I would prefer to come here [the CTC] … when you come here and you see your fellow patients, you laugh together, like you don’t have any other problem and you try to hear other person’s opinions … you find everyone is giving their opinions and laughing so you just feel good.” (PLWH P30)

### Who should provide care and who should receive it?

HCPs consistently noted that the doctors in the CTC and in the OPD are all trained in internal medicine and have the same level of education and skills. Therefore, CTC HCPs could provide care for diabetes and hypertension and OPD HCPs could provide care for HIV; however, it was said that managing diabetes and hypertension is easier than managing HIV. As a result, most HCPs preferred for CTC doctors to provide the care for diabetes and hypertension in an integrated system. Additionally, CTC HCPs already measures blood pressure and blood glucose when equipment is available. Alternatively, it was suggested to have a doctor that specialises in diabetes and hypertension care to provide care within the CTC.

“like testing for diabetes, that test is very easy to conduct, it can be non-lab personnel. So if it can be brought here [to the CTC] and people get updated on how to use, it can help us to identify a lot of patients … As long as you know all the steps to take when testing then it can be done.” (CTC nurse P5)

“those devices that are used for diabetes and hypertension patients, like your BP machine and glucometer and drugs could be transferred here at the CTC so when you finish issues for the CTC, you can go for other conditions to get treatment. But if we say for CTC drugs to go to our clinic, it would be a challenge.” (OPD doctor P20)

“I wish that the doctor from the hypertension clinic could be here at the CTC clinic so when I come here I finish everything … first of all that avoids wasting time and second it gives hope that everything I have is received here.” (PLWH P25)

It was mentioned by a few participants that patients with diabetes or hypertension should not be cared for in the CTC unless they are also living with HIV; this is because they may then stigmatise themselves. It was said that PLWH would not want people without HIV receiving care alongside them due to fear that others will know their HIV status.

“Those that have diabetes or hypertension, they will start to stigmatise themselves, if one has no HIV.” (OPD nurse P16)

“for example, you have diabetes and me here I have HIV, so when you see me, you go outside and spread information with other people. That’s the only thing I think would be a challenge. It would be a challenge to mix the two care … For example, I have my own file and I am known to have that problem, there should be doctors that are specialists on this problem here [at the CTC] but the diabetic clinic should be there as usual… basically when you mix them both here, people with HIV will not be comfortable.” (PLWH P30)

### What care should be provided in an integrated system?

Most HCPs and PLWH said that all services, from screening, diagnosis, prescriptions and follow-up care of diabetes and hypertension should be integrated into CTCs. It was mentioned by a few HCPs that if the CTC and OPD IT system was integrated, the management of diabetes and hypertension could effectively continue at the OPD; however, HCPs and PLWH stated that it would be an issue for someone’s HIV status to be shared with other clinics/HCPs outside the CTC due to fear of stigma.

“As you know the CTC is confidential and it has a lot of secrets so it cannot integrate with the OPD system or the system for other conditions.” (CTC doctor P7)

“instead of that client to use a whole day in the clinic in the CTC and then go to the OPD for NCD clinic because following the lines take a long time, and from the clinic, they have to go to the laboratory and then come back to the doctor’s room again. So if their file is in the CTC, they should get everything in the CTC, they should have a single file and hold the investigation and medication in the CTC instead of the CTC being here and diabetes or hypertension clinic being in the OPD. That is my wish because you can find a client is in many movements and follow many lines.” (OPD doctor P17)

“I think all kinds of services should start here [CTC] and end up here at the CTC clinic because first to know if the patient has hypertension is done here and his records should be here, that this patient has this problem, and that problem and that problem… everything has to start from here [CTC]… because I know if I come here [CTC] I will finish everything here but now you find you go there and there and there” (PLWH P23)

### When should care be provided?

PLWH stated that diabetes and hypertension care should be provided at the same time as HIV care, which can be as few as two appointments a year; HCPs referred to this as well. Some PLWH spoke about how this flexibility encourages them to maintain adherence and it reduces the burden of having multiple visits to the healthcare facility. However, for diabetes and hypertension care, follow-up visits are currently required monthly. Given the preferences for flexibility and the desire to reduce healthcare visits, it was implied that diabetes and hypertension care should be provided on the same basis as HIV care (i.e. based on stability and adherence).

“they normally ask us about our progress and when they take our measurements for our health and if they see my progress is good then they change the medication from one month to three months so this gives even us hope that we are doing well. And then we continue to follow their advice.” (PLWH P22)

“if today is the clinic for diabetes, tomorrow it will be a clinic for people with hypertension. So the client has both diabetes or hypertension, you see that is a disturbance to the client because he or she will come today and also tomorrow.” (CTC doctor P1)

### Barriers and facilitators for integrating care for HIV, diabetes and hypertension

Table 2 summarises the barriers and facilitators mapped to the relevant TDF domains, with supporting quotes for each. Whilst eight domains were initially identified as being relevant, no barriers or facilitators were found for the domain of ‘memory, attention and decision processes’. The most mentioned domain was ‘environmental context and resources’ (16 HCPs contributed toward this domain), with the domain of ‘social influences’ being a close second (n = 15 HCPs). The former domain comprised all barriers whereas the latter had one facilitator and two barriers.

**Table 2:** Barriers and facilitators for integrated care mapped to the theoretical domains.

Theoretical domain	Content mapping (n)	Barriers and facilitators	Supporting quotes
**Environmental context and resources** (Any circumstance of a person’s situation or environment that discourages or encourages the development of skills and abilities, independence, social competence and adaptive behaviour)	16 HCPs in total contributed to this domain	No facilitators were raised under this domain. The barrier most mentioned (n = 8) was regarding the lack of space within the CTC to include care for diabetes and hypertension. Lack of equipment (n = 5) and staff (n = 4) were two additional barriers. It was said that even the OPD does not always have the necessary equipment for diabetes and hypertension. HCPs were worried about the workload that integration would create for CTC staff, stating that they often already struggle with workload. Several HCPs (n = 6), mostly OPD HCPs said that integration would not solve the issue of NCD medications being costly and unavailable. Two HCPs said a guideline is needed to ensure care for diabetes and hypertension among PLWH was done correctly. One HCP said the CTC filing system is currently incapable of keeping track of comorbidities.	*“our CTC building is too small compared to the number of clients that we are attending so if we could have enough building and a specific room for caring for non-communicable disease it would be good*.*”* (CTC doctor P6) *“it would be easier if we had adequate manpower*, *it would be easy to take care of all the patients that are diabetic and give medications to patients that are diabetic*. *Because taking care of these patient you have to have time to talk to them*, *hearing from them expressing their signs and symptoms*, *taking the measurements*, *coming up with the diagnosis and the treatment … the doctor might be overworked because you can’t predict the number of patients a day here at the clinic so you may find you have 100 plus patients … some months you find the patients never stop*. *You write until you feel the pain in the fingers*. *You get tired*, *even*, *you can’t rest*.*”* (CTC doctor P3) *“most of the medication in here [at the CTC] are for free*, *so the challenge I see maybe with the NCD medication*, *they require for them to buy*. *That is the challenge I see*.*”* (OPD doctor P14) *“there must be a guideline that shows that if the patient is having certain comorbidities then there is a certain way to take care of them*.*”* (OPD doctor P12) *“the system needs improved so we can even care for non-communicable diseases*.*”* (CTC doctor P7)
**Knowledge** (An awareness of the existence of something)	11 HCPs in total contributed to this domain	Two facilitators and no barriers were raised under this domain. Seven CTC HCPs knew of specific signs and symptoms of diabetes and hypertension, stating they can identify when someone needs further investigation. Six HCPs expressed their knowledge on contraindications for PLWH and for certain ARTs; both CTC and OPD HCPs were knowledgeable about this.	*“when one can tell you that ‘I feel tired’ and they have frequent urination and they are thirsty and you know these are the signs of diabetes and even their skin is like pale*.*”* (CTC nurse P4) *“a patient with diabetes and HIV they may be changed to second line medication because metformin may interfere with HIV medication*.*”* (CTC doctor P9) *“you may find the HIV has caused problems to their kidney and the treatment regimen has changed from the normal treatment regimen … you may find that the medication be changing due to the complication of another disease for example metformin may be changed because the kidney has failed*.*”* (OPD doctor P12)
**Skills** (An ability or proficiency acquired through practice)	9 HCPs in total contributed to this domain	Six CTC HCPs said they have the skill to diagnose diabetes and hypertension and three said they have the skills to manage PLWH with diabetes and hypertension (facilitators for integration of care). However, six HCPs (including one OPD HCP) said that CTC HCPs need updates and training on best practice for managing diabetes and hypertension among PLWH.	*“I will rely on the symptoms that they present to interpret the results and after doing some investigation because after investigation I will have the results and I must interpret the results*. *That is the skill that I have*. *Now I will know if they are diabetic and if diabetic*, *this is the normal range but this is above the range*. *Then I will know*. *Or for pressure*, *there is a normal range for blood pressure but this one has above the range so I will diagnose by knowing that*.*”* (CTC doctor P1) *“taking for example the blood pressure and finding that it is 140 over 90 or any reading that is abnormal*, *because we know which readings are normal and abnormal so sometimes we may find its 200 over 100 mm/Hg*.*”* (CTC nurse P2) *“if we have the equipment and supplies*, *we could be better*. *But also if we could be given more on job training it would help us to take care of these patients*.*”* (CTC doctor P10) *“the CTC healthcare workers cannot be competent in taking care of NCDs until they practice for a week then they can get used to that*.*”* (OPD doctor P17)
**Professional role** (A coherent set of behaviours and displayed personal qualities of an individual in a social or work setting)	2 HCPs in total contributed to this domain	Two CTC HCPs mentioned one barrier each under this domain: 1) mentorship should be available within the CTC and 2) CTC HCPs must be more committed to helping PLWH with other conditions for integration of care to be effective.	*“But also we need more trainings*, *like mentorship or what*, *it should be done more frequently so that we are not too busy with only HIV*.*”* (CTC nurse P2) *“if we have ourselves as healthcare providers are committed and if we are empowered to get on job training so that we can be more expert*.*”* (CTC nurse P8)
**Optimism** (The confidence that things will happen for the best or that desired goals will be attained)	4 HCPs in total contributed to this domain	Four CTC HCPs mentioned the same facilitator under this domain (no barriers were raised); that is, integration of care at the CTC would bring no challenges and it would be easy to do.	*“if we have the supplies for managing diabetes and hypertension from investigation to medication there is no problem*, *it is possible*.*”* (CTC doctor P1) *“here I am not working alone*, *I’m working as a team*. *We have nurses and doctors here*, *we have social workers*, *we have nutritionists so because we have a team we cannot fail to take care of patient*.*”* (CTC nurse P2)
**Beliefs about capabilities** (Acceptance of the truth, reality or validity about an ability, talent or facility that a person can put to constructive use)	6 HCPs in total contributed to this domain	Six CTC HCPs were confident in their capability to diagnose and manage diabetes and hypertension for PLWH; it was mentioned that as long as the CTC has equipment, medication and trainings on updates regarding treatment, they will be able to provide that service.	*“For example like testing for diabetes*, *that test is very easy to conduct*, *it can be non-lab personnel … Just like it is on HIV that even non-lab personnel conduct those tests*. *As long as you know all the steps to take when testing then it can be done also for a case of diabetes*.*”* (CTC nurse P5) *“I don’t know how [caring for diabetes and hypertension] that could be difficult*. *It’s only a matter of prescription and the patient goes to take the medication*. *Therefore*, *I have that competence because this is my job*.*”* (CTC doctor P9)
**Social influences** (Those interpersonal processes that can cause individuals to change their thoughts, feelings, or behaviours)	15 HCPs in total contributed to this domain	One facilitator and two barriers were raised under this domain. Most HCPs (n = 12) said that family, friends, peers and community support is an enabler for PLWH to attend healthcare appointments and adhere to the medications and lifestyle advise. However, six HCPs said that PLWH may be lost to care and not adhere to medications or instructions for HIV, diabetes and/or hypertension due to influences from society, traditional medicine or non-acceptance of their HIV status. One OPD HCP also said that making PLWH receive care for all comorbidities in one place may make them feel they are being isolated from other clinics which can result in self-stigmatisation.	*“family and friends has a huge contribution because they are the ones that ensure their relative has to receive medication and when he is too weak and can’t take medication so the family and friends that can make sure he takes medication*. *These drugs are very strong*, *you must eat so that they can eat properly so family and friends they make sure they are taking the right amount of food*. *They are the ones that remind that today you have to go to the clinic they remind you have to take medication*. *If they are having 3 different medications therefore it is the closest people that has the largest contribution*.*”* (OPD doctor P19) *“You know speaking with the mouth is easy but when you get the problem*, *it is not that easy*. *You may find that a patient come and reject and deny everything*. *You may find that some patients reject the care and decide to go seek prayer from pastors and some after prayers they come and declare they are negative*.*”* (CTC doctor P3) *“here as a healthcare provider*, *I might be doing the right thing of taking care of the patient*, *but if the patient goes home and is not following what I have told him or her then that won’t be helpful*, *but also the community in which the patient lives*, *if they are not going to accept the situation and starts stigmatisation then the services we provide here won’t be successful*.*”* (CTC nurse P8) *“[traditional medicine] is a big challenge and they come when they are in a very critical stage*, *but there is not so many … So when they come*, *you can find that their kidney is almost failed and we start treatment from scratch*.*”* (OPD doctor P11) *“they can feel there is a stigmatisation of HIV clients*, *that we have isolated them for them not to infect others*. *And even some organisations*, *they may ask*, *why have we isolated these clients*.*”* (OPD doctor P15)

HCPs: healthcare professionals; CTC: care treatment centre; OPD: outpatient department; NCD: non-communicable disease; PLWH: people living with HIV; BP: blood pressure; ART: antiretroviral therapy

## Discussion

We describe HCPs’ and PLWH’s perspectives of how care for diabetes and hypertension can and should be integrated with HIV care. There was a general consensus among participants that diagnoses and management of diabetes and hypertension should be provided for PLWH at the CTCs and this service should be delivered by either CTC doctors or NCD specialists working within the CTC. Based on participants’ preferences for the flexibility of follow-up HIV care and reduced visits to the healthcare facility, follow-up care for diabetes and hypertension should be aligned with HIV follow-up appointments and be provided up to twice a year based on the stability of their condition(s). We applied the TDF to HCP data for a formal assessment of any potential barriers and facilitators for integrating services based on participants’ perspectives. HCPs stressed many barriers for achieving this preferred integration approach, mainly to do with two domains: environmental context and resources, and social influences. However, HCPs mentioned several facilitators regarding HCP knowledge, skills, optimism and beliefs about capabilities.

The strong preference we report for the integration of HIV, diabetes and hypertension care is in line with existing evidence from HCPs and PLWH in South Africa, [15] and from stakeholders in Nigeria [16]. Out of the three models of HIV and NCD integration described by Duffy et al. [8] the preferences from our participants are most related to model 1 (Fig 1); however, our findings highlight some important alternatives within the details of this model. Duffy et al. [8] describes model 1 as involving those with NCDs only and/or PLWH that have NCDs to be cared for in the same clinic. As raised by our participants, PLWH may not feel comfortable with NCD patients receiving care at the same clinic, and NCD patients without HIV may stigmatise themselves. This was evidenced by a ‘one-stop shop’ in Uganda and Tanzania (Dar es Salaam); authors found that NCD patients complained to HCPs about the integration and some PLWH went elsewhere for care, particularly young PLWH [10, 12, 18]. Furthermore, evidence suggest this type of integration would have little to no positive impact on clinical outcomes and adherence [30]. Another component of model 1 described by Duffy et al. [8] was the use of community health workers. Our participants were apprehensive about the use of community health workers due to fear that this would reveal PLWHs’ HIV status to others in the community; this is corroborated by evidence from a South African study [14]. These findings raise the importance of improving patient-centred care delivered through the DSD model to ensure the delivery of care is tailored based on individual preferences. Using the DSD model for integrated care aligns with recognition from the scientific community that applying this model to NCD care has the potential to improve clinical outcomes and adherence to medication [31]. This is particularly the case given the flexibility of follow-up care incorporated in the DSD model which can eliminate critical barriers to care, improve efficiency of care and reduce unnecessary burden on PLWH with NCD comorbidities [31]. Preliminary evidence from Eswatini shows that using the DSD model to integrate HIV and NCD care is effective at improving health outcomes and adherence [32].

Many of the barriers that HCPs mentioned in the current study were also mentioned by stakeholders in Nigeria, including limited equipment, costs of NCD medicine and lack of guidelines [16]. These barriers have consistently been raised in studies assessing services after integration [9, 14, 18] and our previous study reported similar barriers and facilitators of the current care delivery (i.e. fragmented services), [6] indicating that these barriers already exist and would not be generated by integrating care. Indeed, quantitative evidence from across sub-Saharan Africa show that most healthcare facilities lack capacity for diagnosing and managing diabetes and hypertension due to lack of equipment and medicine [33, 34]. In Tanzania, only 33% of health centres have regular access to metformin and only 51% have access to a glucometer with strips [33]. Another major barrier raised by HCPs in our study was the size of and space for adding another service of care at the CTC. However, if only PLWH with and without NCDs are provided care at the CTC (as our participants preferred), integration would not change the number of people receiving care at the CTC; the size and space of CTCs should theoretically be inconsequential in the preferred integration approach.

Family and friends contribute substantially toward the health and wellbeing of people living with chronic conditions; [35, 36] reminding them about healthcare appointments and taking their medication and encouraging them to live a healthy lifestyle. This was raised by our HCP participants as an enabler for integrated care. CTCs provide disclosure counselling to PLWH to encourage them to disclose their HIV status to at least one person; this practice could also positively impact adherence and retention in care for diabetes and hypertension in an integrated system [37]. Traditional medicine was raised by HCPs as a potential barrier for NCD care along with non-acceptance for HIV care; however, these barriers were also raised in our previous research regarding the current delivery of care [6]. Thus, our study highlights that integrating diabetes, hypertension and HIV care are unlikely to overcome these barriers that are already present in a fragmented system. Therefore, irrespective of how care is delivered, more efforts are needed to educate the public on chronic conditions (including diabetes, hypertension and HIV) and improve healthcare seeking behaviours among people with chronic conditions.

Our study has many strengths. First, our 36 participants comprised PLWH with comorbidities of diabetes and/or hypertension, HCPs who provide care for HIV and HCPs who provide care for NCDs. Thus, our sample was diverse and included perspectives from those that would be impacted most by integrating diabetes, hypertension and HIV care. Second, we recruited from the two healthcare facilities with the largest number of registered PLWH in Dodoma, representing both rural and urban inhabitants; therefore, it is likely our results are transferable to the wider region. Third, using the DSD building blocks [7] and TDF [19] as frameworks for presenting preferences and barriers and facilitators for the preferred integrated system allowed for a more focused and meaningful interpretation of data which can be practically applied in future healthcare planning. One major limitation to mention is that PLWH who are not retained in care were not interviewed. Those lost to care may have a different preference of how care should be delivered, particularly if the reason they are lost to care is due to stigma associated with attending CTCs. Additionally, we did not interview people with only diabetes or hypertension; however, this may have provided an important perspective to how care should be integrated. These findings may only be transferable to people of similar demographics, experiences and within similar settings as Dodoma [38].

This qualitative study provides valuable insights into how care for diabetes, hypertension and HIV care should be integrated in sub-Saharan Africa and what barriers and facilitators may impact such integration. Healthcare systems across Africa must improve to accommodate the increased burden of NCDs among PLWH and understanding the perspectives of those most affected by health system changes is necessary for effective implementation. Our study indicates that HCPs and PLWH want all diabetes and hypertension care to be provided alongside HIV care in HIV clinics at the same time and on the same basis as HIV care. The main barriers that HCPs highlighted for this integration approach were barriers that already exist in a fragmented system and many facilitators exist that could aid in making integration effective. These findings should be used to guide decisions on integration of care to achieve optimal care for PLWH with a comorbidity of diabetes and/or hypertension.

## Supporting information

S1 Checklist COREQ checklist. (PDF)

S1 File Inclusivity in global research questionnaire. (DOCX)
